# Moral Distress of Nurses Working in Paediatric Healthcare Settings

**DOI:** 10.3390/healthcare12131364

**Published:** 2024-07-08

**Authors:** Ana Cristina Ribeiro Miranda, Sara Duarte Fernandes, Sílvia Ramos, Elisabete Nunes, Janaína Fabri, Sílvia Caldeira

**Affiliations:** 1 Nursing School Lisbon, Universidade Católica Portuguesa, 1649-023 Lisbon, Portugalseramos@ucp.pt (S.R.); 2 Nursing Research, Innovation and Development Centre of Lisbon (CIDNUR), Escola Superior de Enfermagem de Lisboa, 1600-190 Lisbon, Portugal; enunes@esel.pt; 3 Faculty of Nursing, Universidade Estadual do Rio de Janeiro, Rio de Janeiro 20551-030, Brazil; 4 Center for Interdisciplinary Research in Health, Faculty of Health Sciences and Nursing, Universidade Católica Portuguesa, 1649-023 Lisbon, Portugal

**Keywords:** moral distress, moral resilience, paediatric care

## Abstract

This scoping review aims to map the evidence on moral distress of nurses working in paediatric healthcare settings from homecare to hospital. It was conducted according to the Joanna Briggs Institute. International databases were searched according to the specific thesaurus and free search terms. Independent screening and analysis were conducted using Rayyan QCRI. This review considered a total of 54 studies, including quantitative and qualitative studies, systematic reviews, and grey literature; English and Portuguese languages were included. Moral distress is a phenomenon discussed in nursing literature and in the paediatric context but is considered absent from discussion in clinical practice. It is caused by disproportionate care associated with overtreatment. Nurses can present a variety of symptoms, characterising moral distress as a highly subjective experience. The paediatric contexts of practice should promote a healthy ethical climate and work towards a moral community built with peer support, education, communication, leadership, and management involvement. Moral distress is still a complex and challenging multidimensional concept, and the aim should be to promote a culture of prevention of the devastating consequences of moral distress and work towards moral resilience.

## 1. Introduction

The concept of moral distress was first described by Andrew Jameton in 1984 in a book publication. The inspiration for this work was the nursing students’ experiences in the hospital environment shared in Jameton’s classroom. This author suggests that moral distress causes negative feelings and arises from the impossibility of acting the right way [1].

The moral can be influenced by society’s values, rules, and subjective principles of each professional in a specific time [2]. It allows for the achievement of personal well-being and life sense. In this way, when a nurse acts against his or her own moral, this action becomes immoral. According to Corley, inadequate action happens due to external factors, organisational and social, or due to personal factors, which can lead to moral distress, having a physical, emotional, psychological, and relational impact on one’s own self and others [3,4,5,6].

Moral distress is still a conflicting concept and is supported with weak knowledge [2]. In the same line of thought, the American Association for Critical Care Nurses (AACCN) recognises this as a complex and challenging concept, and the most important difficulty relies on identifying the phenomenon [7].

Fachini et al. [5] identified, in a study with paediatric intensive care nurses, the lack of technological resources and dehumanised care as the causes for poor quality of care and sources for moral distress. Also, the loss of professional autonomy allows for the hierarchy power to lead in action, not professional moral values. Interestingly, moral distress can likewise arise in an inequality of resource distribution locally and worldwide. Later, Passos dos Santos et al. [8] added that end-of-life care with child pain, prolonged suffering, and difficulties in communicating with the child/family and within the healthcare teams are also causes of moral distress identified by nurses working in paediatric intensive care units.

Similarly, moral distress is also frequently explored in paediatric oncology. Nurses identified painful procedures, parents not telling the truth to children, team conflicts poorly dealt with, and lack of human resources and time to fulfil the child and family needs as causes for moral distress. Besides this, authors alert that the intensity of each situation is more detrimental than its frequency [9].

The literature is clearly identifying the devastating consequences of moral distress for nurses, like burnout. This is defined as the emotional, physical, and psychological exhaustion caused by professional activity and leads to avoidance and depersonalisation [6], which may result in poor quality of care, job change, and leaving the nurse profession [7,9].

On the other hand, and more importantly, moral distress can inspire self-reflection, personal growth, and child/family advocacy [4,10].

Health professionals are human, so it is impossible to end moral distress. The focus is to reduce it, allowing for a healthy and respectful nursing career [8].

The main purpose is to achieve moral well-being, which is the coherence between thought and action and highlights the need to develop strategies and resources to resolve moral conflicts [6,9,11].

Although the severe implications that moral distress can have for nurses have already been identified by several authors [10], no review on moral distress in nurses working in paediatric settings has been identified, which reinforces the need to map and organise the available evidence on this concept in this specific population and context. Additionally, Jameton alerts that it is still necessary to develop knowledge around this subject for its incoherencies [1].

As the interaction of the diverse and complex context of paediatric healthcare settings, hunted with moral distress, and the demands for a recognised specialist paediatric nurse can only thrive if the nurse can recognise and deal with self and others’ moral distress. Studying this subject elevates the nurse professional as a moral agent, characterised by the courage needed to advocate for the child/family’s best interests, independently of others’ opinions [11]. Thus, studying moral distress in nurses working in paediatric settings reflects the development to more humanised and realist care for the paediatric population.

In this way, the main objective of this scoping review was to map the available evidence on moral distress for nurses working in paediatric settings from home care to the hospital and, more specifically, to map its definitions, causes, symptoms, consequences, and suggested strategies in paediatric contexts.

## 2. Materials and Methods

This scoping review was conducted according to the guidelines from the Joanna Briggs Institute [12] to answer the following review question: What is the available evidence on moral distress of nurses working in paediatric healthcare settings? Specifically, this literature review aimed at mapping the definitions, the causes, the symptoms, the consequences, and the managing strategies of moral distress in nurses as participants and in paediatrics as the context of care.

According to the JBI guidelines, the inclusion criteria were as follows: Participants—studies that include nurses working in paediatric healthcare settings; Concept—studies related to moral distress, including at least the definition, symptoms, causes, consequences, or strategies; Context—studies that include nurses working in paediatric healthcare settings who care for children/family in a variety of settings from homecare to hospital.

This scoping review considered for inclusion quantitative, qualitative, and mixed methods study designs. In addition, systematic reviews, opinion papers, and a thesis were included.

The search strategy included both published and unpublished primary and secondary studies. An initial search was conducted in Medline and CINAHL using text words related to moral distress.

The text words contained in the titles and abstracts of relevant articles and the index terms used to describe the articles were used to develop a full search strategy with the collaboration of a scientific librarian in May 2022 in the following databases: Pubmed ((Nurses, pediatric[MeSH Terms]) OR (“pediatric nurse*”)) AND ((Morals[MeSH Terms]) OR (Stress Disorders, Post-Traumatic[MeSH Terms]) OR (“moral distress”) OR (“moral suffering”) OR “moral constrain*” OR “moral reckoning” OR (Conscience[MeSH Terms]))retrieving 429 results; Medline ((MH “Nurses, Pediatric”) OR “pediatric nurse*”) AND ((MH “Morals”) OR (MH “Stress Disorders, Post-Traumatic”) OR “moral distress” OR “moral suffering”) with 25 results and CINAHL ((MH “Pediatric Nurse Practitioners”) OR (“pediatric nurse*”)) AND ((MH “Morals”) OR (MH “Stress Disorders, Post-Traumatic”) OR (“moral distress”) OR (“moral suffering”) OR “moral constrain*” OR “moral reckoning” OR (MH “Conscience”)) with 31 results.

Sources of unpublished studies and grey literature were searched in Google Scholar and Institutional Repositorium. All articles in English and Portuguese were considered.

**Figure 1:**
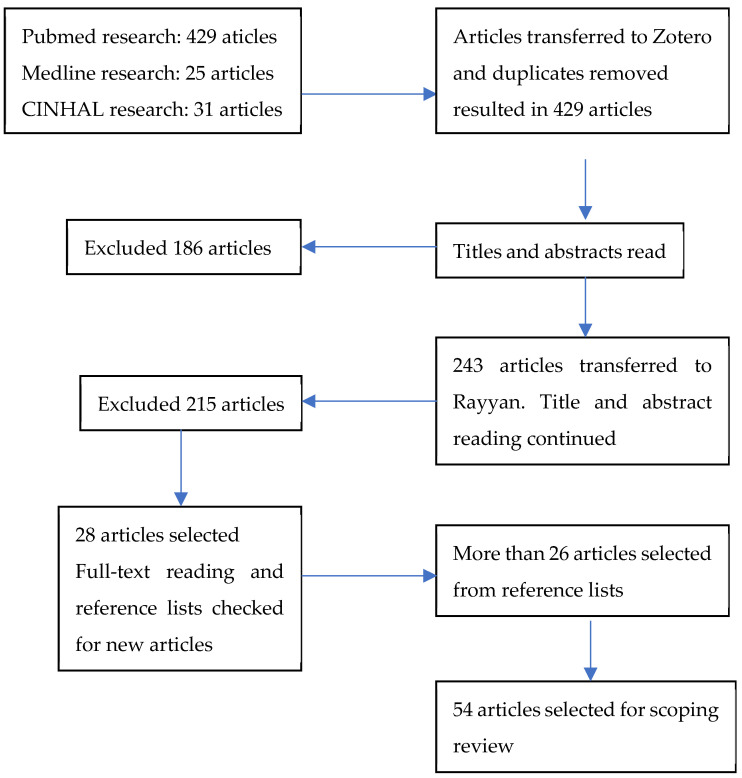
PRISMA-ScR = Preferred Reporting Items for Systematic reviews and Meta-Analyses extension for Scoping Reviews.

Following the search, all identified records were collated and uploaded into Zotero, and duplicates were removed. Following a pilot test, titles and abstracts were then screened by two independent reviewers for assessment against the inclusion criteria for the review. The last screening was performed after transfer to Rayyan to select articles and to facilitate the resolution of conflict among the revisors. A total of 54 articles were selected for the scoping review (Figure 1). The third stage of the search was conducted after the full-text reading when all references of these papers were retrieved for additional analysis.

The potential 54 relevant papers were retrieved, and full-text reading and analysis were conducted, always considering the inclusion criteria, by two independent reviewers. No more papers were excluded from this scoping review. Any disagreements raised between the reviewers at each stage of the selection process were resolved through discussion.

Due to the high number of papers selected, the reviewers firstly created a data extraction tool with the following aspects of each study: author, year of publication, title, country, population, type of study, context, and key findings relevant to answer the review question. Data were extracted from the papers included in the scoping review by two independent reviewers (Supplementary Table S1).

## 3. Results

Different definitions and related concepts of moral distress have been identified (Table 1), and the following attributes were common, such as the suffering caused by an imbalance between actions and one own’s convictions due to internal and/or external factors. More so, moral distress is a subjective experience, and therefore, one’s own perception is considered key to recognise and understand the phenomenon. The tables organise all the results, and the references are according to the Supplementary Materials.

Alongside this, we have also identified the causes, influencing factors, symptoms, and consequences of moral distress and the strategies of dealing with moral distress (Table 2).

**Figure 2:**
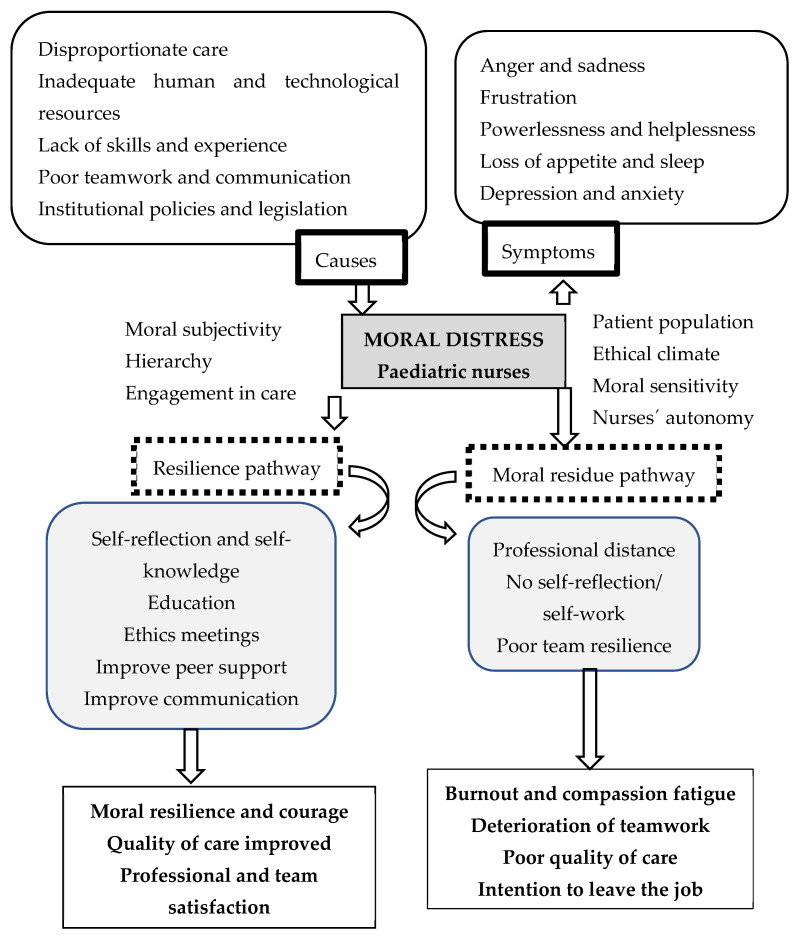
Map of the available evidence on moral distress in nurses working in paediatric care.

Due to the high number of results and the complexity of the subject, Figure 2 summarises the results presented above, working, as well, as an introduction for the discussion chapter.

## 4. Discussion

The main purpose of this scoping review was to map the evidence of moral distress in nurses working in paediatric care.

The mapping of the available evidence related to moral distress disclosed that many of the studies took place in NICU’s, PICU’s, and oncology wards, mainly due to their high-tech and fast-paced environments [13] and because they are the most ethically challenging [14].

Firstly, to clarify moral distress as a concept, definitions were explored, and in the 1990’s terms, moral dilemma and moral distress were interchangeably used [15]. However, today, the concepts are distinct; one questions the course of action between two acceptable options (moral dilemma), while the other happens due to constraints on taking the right (already chosen) course of action [16]. Moral distress surfaces when medical decisions or plans of care challenge one’s personal and moral beliefs [17,18]. Moral distress relates to the suffering that affects the body, mind, and relationships when one is clearly aware of what should be done but, due to the circumstances, is unable to act accordingly [5].

Secondly, moral distress can present in two forms, as initial and reactive/residue, the former presenting as symptoms, and the latter feeling as if one did not act on the initial distress [19,20]. Another finding is the moral distress conceptual model in which two pathways are available. On one side, the nurse is empowered to be the moral agent, to act and confront the situation, coping with it as an advocate for her/himself and others and allowing for a positive outcome, such as moral resilience. On the other side, the nurses face ‘stagnation-in-uncertainty’. The continuous sense of powerlessness leads to inaction and resignation, which can be comprehended as the norm, creating challenges to advocacy and leading the way to passivity and moral residue [21,22].

Additionally, several pieces of literature were found concerning related concepts to moral distress. The nurse, seen as a moral agent, is the way forward to a positive outcome and implies moral courage to do what one knows as the correct course of action [8,14,23,24]. For this to happen, each nurse needs to develop moral sensitivity, perceived as an acquired skill to develop moral action and competence [21,22,25,26,27]. Concomitantly, an ethical climate that focuses on the interactions between the health professional and the surrounding organisational structures can be a healthy promotor or an oppressive factor for ethical situations to be identified, discussed, and decided [16,27,28].

The causes for moral distress are a recurrent theme across most of the studies and, in some studies, already divided by categories: family-centred, team-focused, or regarding management, and, in other studies, listed directly by the participants. One cause, often agreed on by professionals, was disproportionate care related to overtreatment, especially when investing one’s energy, knowledge, and time as well as technological and material resources to prolonging someone’s life when the professional ‘following orders’ believed it to be futile [16,17,29,30,31]. This idea is well-represented by Silverman’s expression, “Once on the train, it is hard to get off” [32] (p. 293). And it is related to the different perspectives of care between nurses and physicians discussed by several authors [21,33,34].

Intimately related to this cause is the related concept of hierarchy and its oppressive effects on team members to participate in the decision-making process [29,35]. Besides this reality in intensive care units and oncology wards, in mental health, disproportionate care was more related to using extreme measures of isolation and de-escalation of disturbing children’s behaviours [36].

Poor communication is the main source of conflict, or even the lack of it, between professionals and the patients/families regarding difficult topics, like death [8,16], reports of child abuse, and poor articulation between community services [15]. It is associated with poor multi-disciplinary team collaboration and a negative impact on the quality of care [37], for example, the families’ frustration regarding the quality of the communication and the dialogue involving the diagnosis, prognosis, and care plan [38,39].

Communication has an impact on the ethical climate, as open conversation channels within the team promote reflection and discussion [40]. Instead, silenced nurses’ voices, also identified as one of the causes and characterised by nurses’ voices being unheard or their opinion ignored not only by colleagues and families but also management, decrease professionals’ insight and the possibility for their own self and team growth [8].

Staffing levels were another recurrent cause mentioned by nurses, resulting in a reduced amount of time spent with the patient and family, the levels of moral distress gained, and its consequent impact on the quality of care [21,35]. Moreover, results show omitted nursing tasks and missed care due to under-resourced staffing [21].

Considering these studies mostly took part in NICUs and PICUs, which are well-known for their fast-paced routine and demanding expertise, the shortage of nurses can be understandably perceived as one major reason to cause moral distress. Nonetheless, having been identified as a cause, it still needs to be addressed [9,27,29].

As a cause of moral distress, pain management was specifically felt and voiced by the nurses, professionals 24/7 by the bedside, and the ones to provide treatment as of foremost importance. The challenges are getting physicians on board, implementing treatment once it is agreed upon, and then being faced with the dilemma of possibly hastening death [8,30,41,42]. This is related to the educational needs for end-of-life care and pharmacological management [30,40,43].

Furthermore, working with less-skilled nurses or physicians in the context of intensive care units, marked by fast-paced and expertise demands, was stated as a source of moral distress [21,24,27]. For example, in the face of an emergency, a senior nurse will have to compensate for the junior colleague, being confronted with this duality of knowing that the new nurse needs to learn the skills. However, the child deserves their best chances of survival and care [44]. Additionally, the frequency and intensity of moral distress were both high when nurses recognised the inadequate levels of competence in their colleagues [27,45].

The literature highlighted a wide range and diversity of symptoms associated with moral distress. It is a multidimensional experience [21] that can have a physical, emotional, psychological, and relational impact on self and others [4]. In this way, the moral distress phenomenon is marked by subjectivity [45], and here lies one of the reasons it is so difficult to recognise. The process starts inside the nurse professional who is involved in the experience and depends on the ability of one’s own self to identify the emersion of feelings and sensations that appear.

More so, the data highlights the importance of moral sensitivity [22,25], not only from one’s own self but also within the team of nurses and other health professionals including management of each context of practice, to be able to recognise when someone is struggling [27]. And that is why the ethical climate [39] and the hierarchy present in each context influence the experience of moral distress.

Several strategies were identified to mitigate moral distress and determine when they should be implemented [13,17,20,33,46,47]. Due to the human nature of nursing professionals and the high subjectivity of the moral distress phenomenon, it is not possible to eradicate it [8], and a culture of prevention is the most common guidance considering the devastating consequences moral distress can cause [41,48,49,50]. Thus, strategies will be discussed before consequences [5,8,42], as moral distress demands action and moral work [51] to aim for moral resilience.

More specifically, two pathways should be considered when moral distress occurs [22]. The consequences result from the pathway chosen by the nurse professional and the healthcare team involved. This process is intrinsically dependent on everyone’s involvement, from the bedside nurse to the hospital director, highlighting once more the importance of an ethical climate [19,20,25] and potentiating the significant role of nurses as moral agents [8,14,23,24]. More so, it demands moral responsibility [26] and the need for nurses to believe and develop their professional autonomy, which is crucial to be part of the decision-making process [5,17,25,52].

Then, the positive pathway comprises self-reflection and self-knowledge by providing objective questions like the following: “What is it about this situation that is causing me moral distress?”, “Does everybody think the way I do?”, “Can the factors leading to moral distress be modified?” and “How can I voice my concerns most constructively?” [26] (p 42–43). This reflection process builds moral sensitivity and helps to elaborate a justified position when raising concerns with others. Personal opinion is limitative and insufficient to stir the waters when moral distress issues are involved, and it is crucial to be aware that others may think differently [17]. As so, building resilience is mandatory to accept that, within a team, we may need to agree to disagree [47].

Literature underlines the need to build moral work within the healthcare teams by improving peer support [9,20,53,54], like the elaboration of a nurse education and support team coach with positive feedback from nurse professionals [48], developing educational moments related with ethics [37,55], pain management in end-of-life situations [30,40,43], and communication skills [56]. Improving communication skills highlights the need to respect a place for everyone that is part of the team to voice out their concerns [29,34,44]. Agreement among professionals does not always lead to a high quality of care, so discussion of different perspectives must occur [57]. Still, regarding communication to promote informal moments for storytelling is imperative, as sharing with others relieves the burden of moral distress and helps to find answers [13,24,51]. An example of this is some of the articles included in this scoping review of case studies and lived experiences in practice [20,32,47,58].

Lastly, regarding educational needs, nurses identified a lack of knowledge and skills when dealing with specific situations, such as neonatal abstinence syndrome [14], resuscitation of extremely premature infants [49], end-of-life care for the patient and family [41], and pain relief in children with a persistent vegetative state [59].

The main cause of moral distress is related to disproportionate care, which is intimately related to patient care, communication conflicts with the family members, and the controversial weight parents have in the decision-making process. Nurses have doubts if the parents receive all the information needed regarding outcomes and prognosis [30], and the literature is still dubious of whom should decide the treatment options, parents or physicians [47].

So, it is crucial to improve the communication with families by providing realistic and fair information in regular meetings with the team members [46], improving their involvement in the daily care [60] and providing adequate counselling and emotional support [20]. If there are conflicts between the team and the family regarding the decision-making process, additional support should be requested and developed [47]. Interestingly, Woods identified the possibility of nurses’ moral distress reflecting parents’ distress [31]. This is not studied further in the literature and is considered a gap and an opportunity for future research.

Management teams should be role models for all that has been discussed so far. They should promote a healthy ethical climate with strong leadership and give the tools necessary to develop all the strategies mentioned above to prevent the negative consequences of moral distress [29].

The development of ethics committees comes, as well, as a strategy to offer support and understanding when ethical issues have risen within the team and/or between the team and family [8]. Although, it can be concluded from this analysis that the strategies needed to mitigate the negative consequences of moral distress are complex [29,47]. It is well-recognised as the way to reach moral resilience [26,38,61] and moral courage [8], professional satisfaction, and staff retention [21], and, more importantly, to improve the quality of the care provided [20,29].

Unfortunately, this is not the only option; the second pathway is the moral residue pathway, characterised by not dealing properly with moral distress and carrying this weight throughout the daily practice and throughout the nursing career [22,26,49]. In opposition to what was described in the resilience pathway, it is believed there is no self-reflection or no recognition of a moral concern [18] or nurses avoid embarking on moral distress issues, which leads to powerlessness and is accepted as a normal behaviour in many contexts [22]. Poor team resilience and rigid hierarchical contexts [35,45] will have devastating consequences. Related to hospital management, it is recognised that moral distress can be one of the causes to increase intention to leave the job or even the profession, causing the frequent turnover of staff [27,41,46,62,63]. More so, it can have a huge impact on society, as the staff looking after sick children and their families cannot provide high-quality care because they are sick themselves [26,51].

## 5. Limitations of This Study

The authors consider a limitation the exclusion of a few articles due to the impossibility of access to the abstract and even the full text, which could have influenced the analysis of findings.

## 6. Conclusions

Moral distress is a complex and challenging phenomenon that is intrinsically part of nursing. It can result in devastating consequences when the nurse and the multi-professional team do not have or cannot develop the necessary means to mitigate it. Instead, it can be a harsh but well-rewarding process that leads to questioning and reflection of daily practices that results in the development of moral competencies in each team member. In this way, it can create a working environment where everyone who is part of it can voice their concerns, ultimately leading to increased job satisfaction, personal growth, and improved quality of patient care.

This scoping review is another reminder in the literature of how important it is to look after those who care for others and frequently are suffering in silence.

Overall, this literature analysis confirmed the results from previous studies in a more organised and characterised manner. Due to their specificities, it highlights that further and recent research is needed in other paediatric contexts like mental health and community services. More studies need to be developed in practice so the development of strategies for each context happens within the teams and by the teams.

## Figures and Tables

**Table 1:** Definitions and related concepts of moral distress of nurses working in paediatric settings.

Definitions of Moral Distress	
Moral distress	13, p. 58; 55, p. 117; 38, p. 98; 39, p. 102; 15, p. 187; 48, p. 96; 5, p. 114; 53, p. 885; 43, p. 180; 17, p. 365; 20, p. 13; 42, p. 1; 18, p. ii; 14, p. 281; 26, p. 706; 22, p. 500
Moral distress (conceptual models, process)	19, p. 244; 20, p. 13; 51, p. 38; 9, p. 1069; 22, p. 501–502
Positive vs. negative view of moral distress	18, p. iv; 21, p. 2353
Related concepts of moral distress	
Nurse as a moral agent	14, p. 281; 23, p. 111; 8, p. 226; 24 p. 1573
Moral angst	58, p. 607; 31, p. 613
Moral comfort	25, p. 153–154
Moral community	27, p. 44
Moral courage	38, p. 102; 8, p. 229
Moral experiences	21, p. 2359
Moral integrity	26, p. 42
Moral responsibility	26, p. 41
Moral sensitivity	25, p. 153; 21, p. 2352; 26, p. 43; 27, p. 44; 22, p. 500
Moral subjectivity	45, p. 669; 26, p. 41
Ethical climate	39, p. 102; 25, p. 153; 33, p. e20; 19, p. 245; 28, p. 688, 700; 21, p. 2352, 2359; 16, p. 706; 26, p. 43; 52, p. 291; 9, p. 1061–1069; 29, p. 55
Moral competence	55, p. 112; 38, p. 103; 43, p. 186; 21, p. 2359; 51, p. 37
Ethical memory	55, p. 118
Ethical numbness	35, p. 546

**Table 2:** Causes, influencing factors, symptoms, consequences, and suggested strategies of moral distress of nurses working in paediatric settings.

Causes of Moral Distress	
Mental health related	36, p. 41–42
Disproportionate care	5, p. 114; 8, p. 112; 13, p. 61; 16, p. 4; 17, p. 362; 19, p. 245; 20, p. 14; 23, p. 228; 25, p. 150; 27, p. 38; 29, p. 62; 30, p. 734; 31, p. 613; 33, p. e24; 34, p. 586; 41, p. 191; 42, p. 4; 43, p. 181; 45, p. 668; 47, p. 702; 52, p. 289; 59, p. 443;
Team-centred reasons	9, p. 1068; 24, p. 1570; 44, p. e305; 47, p. 4; 49, p. 56
Family-centred reasons	17, p. 366; 22, p. 502; 35, p. 547; 39, p. 105; 47, p. 4–5; 49, p. 55;
Difficulties in communication	8, p. 228; 20, p. 14; 24, p. 1575; 26, p. 702; 33, p. e24; 38, p. 100; 39, p. 106; 40, p. 256; 42, p. 4; 28, p. 692; 53, p. 885;
Inadequate levels of staff	9, p. 1068; 20 p. 14; 21 p. 2358; 27, p. 38; 29 p. 55, 59; 33, p. e24; 35, p. 545; 41, p. 191; 53 p. 885
Inadequate pain management	8, p. 228; 30, p. 735; 41, p. 190; 59, p. 443
Inadequate use of technological resources	5, p. 114; 16, p. 702
Patient-centred reasons	16, p. 706; 47, p. 4; 49, p. 57
Institutional policies and legislation	9, p. 168; 29, p. 56; 53, p. 885
Recognition of lack of skills	14, p. 285; 45, p. 668
Nurses’ silenced voices	5, p. 117; 29, p. 55; 34, p. 586; 35, p. 547; 39, p. 107; 44, p. e306; 52, p. 291
Community health related	15, p. 185
Working with less-skilled colleagues (physicians or nurses)	8, p. 228; 20, p. 14; 21, p. 2358; 24, p. 1570; 27, p. 38; 41, p. 191; 44, p. e306; 45, p. 668; 46, p. 5
Influencing factors	
Autonomy	5, p. 115; 17, p. 361; 25, p. 153; 52, p. 289
Hierarchy	20, p. 14; 23, p. 111; 24, p. 1576; 29, p. 55; 35, p. 547; 42, p. 6
Engagement in care	5, p. 116; 19, p. 245; 43, p. 185
Different goals of care between nurses and physicians	33, p. e24; 34, p. 577
Nurses’ cultural background	35, p. 542
Nurses’ previous bad experiences	61, p. 207
Quality of life	17, p. 363; 39, p. 105
Working night shifts	28, p. 695
Years of nursing experience	42, p. 8
Patient population	5, p. 115
Symptoms	
Ambivalence	22, p. 502; 60, p. 654
Anxiety	24, p. 1570
Anger	5, p. 114; 21, p. 2352; 41, p. 183; 45, p. 664; 53, p. 885; 60, p. 654
Avoidance	18, p. iv
Black humour	18, p. iv; 21, p. 2352
Depersonalisation	22, p. 500
Distress	22, p. 502; 24, p. 1570
Failure	42, p. 1
Feeling frustrated	21, p. 2352; 22, p. 502; 41, p. 183; 42, p. 1; 53, p. 885; 60, p. 654
Feeling helpless/powerless	16, p. 702; 21, p. 2352; 24, p. 1570; 40, p. 255; 41, 688; 28, p. 688; 58, p. 610; 60, p. 654
Guilt	5, p. 114; 21, p. 2352
Isolation	40, p. 255
Lack of commitment	24, p. 1570
Loss of self-worth and devalued	21, p. 2352; 40, p. 255; 41, p. 688
Loss of appetite	39, p. 102
Mortification of interests	22, p. 502
Personal agony	43, p. 180
Poor sleep	39, p. 102
Remorse	21, p. 2352
Sadness	5, p. 114; 60, p. 654
Weight of responsibility of being a nurse	40, p. 255
Consequences	
Burnout	18, p. iv; 21, p. 2352; 25, p. 153; 48, p. 96; 42, p. 2; 28, p. 688
Compassion fatigue	22, p. 500; 50, p. 3
Deterioration of teamwork	20, p. 14; 22, p. 502
Intention to leave workplace or profession/Increased staff turnover	21, p. 2353; 27, p. 35; 41, p. 189; 45, p. 670; 46, p. 5; 51, p. 42; 28, p. 694; 62, p. 47
Lack of job satisfaction	21, p. 2352
Moral residue	16, p. 707; 21, p. 2352; 22, p. 502; 26, p. 41; 49, p. 58
Moral resilience/work	20, p. 14; 22, p. 502; 26, p. 42; 38, p. 101–102; 48, p. 96; 51, p. 39–40; 57, p. 201
Poor quality of care	20, p. 14; 21, p. 2352; 22, p. 502; 26, p. 41; 51, p. 42
Professional distance	26, p. 41; 43, p. 181
Transference of moral distress to others	26, p. 41
Suggested strategies	
Education	8, p. 229; 13, p. 63; 22, p. 503; 23, p. 112; 27, p. 44; 33, p. e23; 38, p. 101; 40, p. 256; 43, p. 187; 48, p. 97; 55, p. 118; 60, p. 656
Multidisciplinary ethics meetings	8, p. 230; 27, p. 43; 29, p. 60; 33, p. e23; 37, p. 78
Ethics committees	8 p. 230; 17, p. 366; 18, p. v; 27, p. 45; 53, p. 886
Peer support/moral community/supportive ethical climate	5 p. 117; 8, p. 230; 9, p. 1069; 18, p. v; 23, p. 110; 24, p. 1575; 26, p. 43; 47. p. 8; 48, p. 96; 28, p. 698; 54, p. 1138
Management-related strategies (staffing, leadership, policies)	8, p. 230; 29, p. 65
Focus on moral strength instead of powerlessness/work towards moral resilience/moral action	20, p. 14; 21, p. 2359; 22, p. 502; 26, p. 42; 38, p. 101; 51, p. 39
Continuity of care	29, p. 55
Spirituality	25, p. 153
Improve communication with parents/Promote meetings with the family	13, p. 63; 20, p. 15; 46, p. 6; 55, p. 119; 60, p. 656

## Data Availability

The original contributions presented in this study are included in the article/Supplementary Materials; further inquiries can be directed to the corresponding author/s.

## Supplementary Materials

The following supporting information can be downloaded at: https://www.mdpi.com/2227-9032/12/13/1364/s1, Table S1: Extraction data of each paper included in the review.
