# Supplementary material for: Moral Distress of Nurses Working in Paediatric Healthcare Settings
Source: Healthcare (Basel). 2024 Jul 8;12(13):1364. doi: 10.3390/healthcare12131364 (PMC11487391; doi:10.3390/healthcare12131364)
Supplement: Supplementary file 1 [file healthcare-12-01364-s001.zip › healthcare-3013136-supplementary.pdf]

Supplementary material:

Table S2. DATA EXTRACTION TOOL.

Author/Title	Country	Study	Context
5 Moral distress in PICU	Brazil	Qualitative study	PICU
8 The difficult decisions in paediatric care and moral distress in intensive care unit	Brazil	Literature review	PICU
9 Ethical climate and moral distress in paediatric oncology nursing	Finland	Quantitative study	Pediatric oncology
13 An overview of moral distress and the paediatric intensive care team	Canada	Literature review	PICU
14 NICU Nurses´ Lived Experiences: caring for infants with neonatal abstinence syndrome.	USA	Phenomenological study	NICU
15 Ethical conflicts experienced by certified pediatric nurse practioners in ambulatory settings	USA	Survey questionnaire	Ambulatory context
16 Moral distress within neonatal and paediatric intensive care units: a systematic review	Australia and Canada	Literature review	NICU and PICU
17 Ethics in pediatric end-of-life care: a nursing perspective	USA	Literature review	Pediatric end-of-life care

18 Moral distress in the pediatric intensive care unit: the impact on pediatric nurses	Canada	Case studies	PICU
19 Moral distress and providing care to dying babies in neonatal nursing	Australia	Literature review	NICU
20 Moral distress: A case study	USA	Case study	PICU
21 Moral distress in paediatric oncology: Contributing factors and group differences	Sweden	National study<br>Swedish moral distress Scale-revised	Paediatric Oncology
22 Moral distress of nurses surrounding neonatal abstinence syndrome: Application of a theoretical framework	USA	Literature review<br>Barlem and Ramos theoretical model of moral distress	
23 Moral experiences and moral distress in nurses who care for children with special needs	Brazil	Thesis - qualitative study	Special needs children
24 The moral experiences of paediatric nurses in Brazil: engagement and relationships	Brazil	Qualitative study (narrative interviews)	3 nurses from paediatric ward; 3 PICU; 3 NICU
25 Moral distress in Neonatal Intensive Care Unit RNs	USA	Descriptive, correlational study<br>Moral distress Scale Neonatal-Pediatric Version	NICU

26 The use and misuse of moral distress in neonatology	Australia	Case study	NICU
27 Assessing and addressing moral distress and ethical climate Part II	USA	Moral distress Scale - Paediatric version and Hospital Ethical Climate Survey	
28 Caring for victims of child maltreatment: Pediatric nurses´ moral distress and burnout	USA	Cross-sectional correlational study	Paediatric level I trauma center
29 Organizational influences on health professionals´ experiences of moral distress in PICU´s	Canada	Analysis of a national PICU´s study	PICU
30 Ethical issues related to caring for low-birth-weight infants	USA	Phenomenological study	NICU
31 Case commentary: Baby John	New Zealand	Case commentary	
32 Proximate moral anguish	USA	Opinion paper	NICU
33 Appropriateness of care and moral distress among neonatal intensive care unit staff: repeated measurements	Netherlands	Moral Distress Scale-Revised Neonatal-Pediatric version	NICU
34 Moral obligations of nurses and physicians in neonatal end-of-life care	USA	Qualitative study	NICU
35 Pediatric nurses´ ethical difficulties in the bedside care of children	South Korea	Phenomenological study	Paediatric ward
36 Ethical voices of Pediatric Mental Health Nurses	USA	Etnographic study	Pediatric Mental Health

37 Pediatric Ethics and Communication Excellence (PEACE) Rounds decreasing moral distress and Patient Length of stay in the PICU	USA	Moral distress Scale Revised<br>Moral Distress Thermometer	PICU
38 Moral distress in end-of-life situations	Brazil	Hybrid model of concepts development	PICU
39 Moral distress defined and described by Neonatal and pediatric Critical Care Nurses in a Quaternary Care Free-Standing Pediatric Hospital	USA	Qualitative descriptive study with focus groups	PICU and NICU
40 Exploring moral distress in pediatric oncology: a sample of registered practitioners	USA	Qualitative study	Pediatric Oncology
41 Moral distress in nurses in oncology and haematology units	Italy	Descriptive correlational study<br>Moral distress Scale- paediatric version	Pediatric Oncology and Haematology
42 Moral distress in PICU and Neonatal ICU Practitioners: A Cross-Sectional Evaluation	Canada	Cross-sectional survey	PICU and NICU
43 It´s agony for us as well: neonatal nurses reflect iatrogenic pain	Australia	Questionnaire	NICU
44 A qualitative Study exploring moral distress among Pediatric Resuscitation Team Clinicians: Challenges to Professional Integrity	USA	Qualitative exploratory study	PICU
45 Moral distress in Iranian pediatric nurses	Iran	Cross-sectional study	Various wards
46 Moral distress in the Pediatric Intensive Care Unit: An Italian Study	Italy	Cross-sectional questionnaire survey	PICU

		Moral-distress Scale Neonatal-Pediatric Version	
47 Moral distress in neonatology	Australia	Longitudinal study (18 months)	NICU
48 Building moral resilience through the nurse education and support team initiative	USA	Survey with 2 evaluation periods	PICU
49 Moral distress in the resuscitation of extremely premature infants	Canada	Secondary qualitative analysis	NICU
50 Moral distress and compassion fatigue in nurses of neonatal intensive care unit	Iran	Descriptive correlational study	NICU
51 Moral distress in perinatal nursing	USA	Literature review	Perinatal Nursing
52 The meaning of being ethically difficult care situations in paediatric care as narrated by female Registered Nurses	Norway	Phenomenological study	Various paediatric wards
53 Moral distress in Pediatric Intensive Care	Canada	Literature review	PICU
54 Dilemmas in practice. Keeping a promise.	USA	Case study	Pediatric Oncology Ward
55 Ethical dimensions of paediatric nursing: A rapid evidence assessment	Italy	Literature review	Several contexts

56 Moral Distress in Pediatric Intensive Care Nurses: Experiences with the Death and Dying of Child Patients	Canada	Qualitative study	PICU
57 Moral distress and ethical confrontation: problem or progress?		Opinion paper	
58 Case study: Baby John-nursing reflections on moral angst	USA	Case study	
59 The experiences of pediatric nurses caring for children in a persistent vegetative state	USA	Phenomenological study	
60 Caring for dying infants: experiences of neonatal intensive care nurses in Hong Kong	Hong Kong	Qualitative exploratory study	NICU
61 Moral distress in the neonatal intensive care unit: caregiver´s experience	Canada	Questionnaire	NICU
62 Moral distress and intention to leave: a comparison of adult and paediatric nurses by hospital setting	USA	Descriptive correlational survey	PICU, NICU and non-ICU
63 Caring for dying children: assessing the needs of the pediatric palliative care nurse	USA	Literature review	NICU, PICU, oncology ward
